# Improvement of quality of care for gestational syphilis in the municipality of Rio de Janeiro

**DOI:** 10.11606/s1518-8787.2021055002534

**Published:** 2021-06-07

**Authors:** Brena Gabriella Tostes de Cerqueira, Eliane Pereira da Silva, Zenewton André da Silva Gama

**Affiliations:** I Universidade Federal do Rio Grande do Norte Centro de Ciências da Saúde Programa de Pós-Graduação em Gestão da Qualidade em Serviços de Saúde NatalRN Brasil Universidade Federal do Rio Grande do Norte. Centro de Ciências da Saúde. Programa de Pós-Graduação em Gestão da Qualidade em Serviços de Saúde. Natal, RN, Brasil; II Universidade Federal do Rio Grande do Norte Centro de Ciências da Saúde Departamento de Medicina Clínica NatalRN Brasil Universidade Federal do Rio Grande do Norte. Centro de Ciências da Saúde. Departamento de Medicina Clínica. Natal, RN, Brasil; III Universidade Federal do Rio Grande do Norte Centro de Ciências da Saúde Departamento de Saúde Coletiva NatalRN Brasil Universidade Federal do Rio Grande do Norte. Centro de Ciências da Saúde. Departamento de Saúde Coletiva. Natal, RN, Brasil

**Keywords:** Syphilis, Pregnancy, Quality Improvement, Quality of Health Care, Primary Health Care

## Abstract

**OBJECTIVE:**

To analyze the effect of a multifaceted intervention in the care of pregnant women with syphilis in primary health care.

**METHODS:**

This is a quality improvement project performed in 26 basic care units in the municipality of Rio de Janeiro, between January and December 2017. It has a quasi-experimental mixed design, with previous, later and time series analyses. We evaluated the care provided to all pregnant women with syphilis whose prenatal care that ended during the studied period, using ten quality criteria and one indicator. The intervention was multifaceted, covering permanent education, improvement of records and information systems, audit and feedback, patient education, organizational changes and work processes. We estimated absolute and relative improvements of the criteria and their statistical significance (α = 5%). The facilitators and hinders of the intervention were analyzed according to the Model for Understanding Success in Quality.

**RESULTS:**

After the intervention, there was a total absolute improvement of 6.7% (64.4% versus 71.0%) and relative of 28.8% (p > 0.05). Eight of the ten quality criteria had an improvement, which was significant in four of them (p < 0.05). The monthly indicator of adequate treatment also improved (p < 0.05), but maintained low performance throughout the project. The increase in the compliance of the treatment scheme with the protocol (91.4% versus 99.1%) positively stood out, but the main opportunities for improvement were testing (42.8% versus 48.5%) and treatment of sexual partnerships (42.8% versus 44.2%). Regulatory pressures to improve the monthly indicator and the political-economic crisis experienced by the municipality modulated the effect of the intervention.

**CONCLUSION:**

The project was useful to identify priorities and guide interventions to improve the quality of care for syphilis, although there is still ample room for improvement. The identified problems, as well as the contextual modulators of the effect, should be considered in future interventions.

## INTRODUCTION

The increased incidence of syphilis in several countries of the world has been treated as a serious public health problem. This epidemiological scenario is a reality in Brazil^1^, since the incidence rates of congenital and gestational syphilis almost tripled between 2010 and 2016, from 2.4 to 6.8 and from 3.5 to 12.4 cases per thousand live births, respectively^2^. More recent data from the Ministry of Health indicate that the same rates in 2018 were already 9.0 and 21.4, continuing the growth trend^3^.

Preventing the vertical transmission of syphilis is a priority of the Brazilian Ministry of Health, aligned with the Pan American Health Organization (PAHO)^4^. In 2016, a report by the PAHO^5^ with data from the countries and territories in Latin America and the Caribbean, pointed to the need to strengthen the actions of the strategy and the action plan started in 2010 in order to eliminate the mother to child transmission of HIV and syphilis, since the goals set in 2015 had not yet been reached for all of them, including: a reduction in the rate of mother to child transmission, and HIV incidence (≤ 2% and not more than 0.3 per case per thousand live births, respectively); reduction of the incidence of congenital syphilis (0.5 case per thousand live births); expansion of the coverage of the prenatal care with the at least one consultation (≥ 95%); expansion of coverage of HIV counseling and testing for HIV and syphilis in pregnant women (≥ 95%); expansion of the coverage of treatment with antiretrovirals in pregnant women living with HIV (≥ 95%); and increased coverage of treatment with penicillin in pregnant women with syphilis (≥ 95%)^6^.

A national initiative that targets the fight against syphilis is the *Agenda de Ações Estratégicas para Redução da Sífilis Congênita no Brasil* (Agenda of Strategic Actions for the Reduction of Congenital Syphilis in Brazil)^4^, launched in 2016, which establishes priorities for qualifying health care, as well as the sharing of responsibilities. In addition, in 2017, the *Projeto Resposta Rápida à Sífilis nas Redes de Atenção* (Rapid Response to Syphilis in Care Networks Project) stands out, which seeks to reduce acquired syphilis in pregnant women and eliminate congenital syphilis in Brazil by strengthening and developing strategic actions of universal coverage and actions with priority municipalities, selected by epidemiological criteria^7^.

Studies indicate that late onset of prenatal care, low number of consultations, lack or delay in diagnosis, inadequate or incomplete therapeutic regimen, non-treatment of sexual partnerships and lack of knowledge of the pregnant woman about the disease are the main failures related to the inadequate management of gestational syphilis^8^. Early diagnosis and timely and adequate treatment of pregnant women and sexual partners result in a reduction in morbidity and mortality associated with vertical transmission, and are considered markers of the quality of prenatal care^4^.

In 2018, among the Brazilian states, Rio de Janeiro stood out with the highest rates of gestational and congenital syphilis, concentrating 28.4% of all deaths from congenital syphilis in children under one year of age in Brazil^3^. Among the capitals, the city of Rio de Janeiro had the second highest rate of syphilis detection in pregnant women in the country, with 51.5 cases per thousand live births, and an incidence rate of congenital syphilis of 13.4 — higher than the national average of 9.0, and well above the goal established by the World Health Organization (WHO)^3^, of less than 0.5 cases per thousand live births.

Addressing this issue is a great challenge for Brazilian municipalities, but the use of quality management methods and tools can be fundamental for an organized response to the problem. Among the quality management processes (quality planning, quality monitoring and improvement cycles)^11-13^, improvement cycles make it possible to obtain results for complex problems in a relatively short time, allowing to evaluate the problem and cyclically test changes that can be monitored by statistical control^14^.

Given the above, this study sought to evaluate and improve the quality of gestational syphilis care, in the scope of primary health care, in one of the areas with the highest incidence of syphilis in Brazil. Specifically, the project aimed to: (1) evaluate the quality of care provided to pregnant women diagnosed with syphilis, from the measuring of quality criteria and indicators; and (2) analyze the effect of a multifaceted intervention to improve quality of care.

## METHODS

This is a quality improvement project with quasi-experimental study design, without control and mixed groups, combining time series analysis and previous and subsequent evaluations^15^. It was developed in the city of Rio de Janeiro, in the basic health units (UBS) of the Planning Area 3.3, which includes the second largest population contingent in the city, is located in the north of the city and brings together 29 neighborhoods – many of them with the lowest human development indexes (HDI) and highest rates of urban violence in the municipality^16,17^. During the development of the study, this area had 31 UBS and 180 family health teams, which corresponded to approximately 70% coverage by the Family Health Strategy (FHS) model.

For the purposes of sampling definition, units that did not use electronic medical records or that had been inaugurated in the last six months were excluded. Therefore, 26 UBS were included in the study (154 family health teams).

The improvement intervention was developed following a quality improvement cycle model^11^. On the steps of the design improvements were: identifying and prioritizing improvement opportunities participatively, and based on criteria, using the technique of a nominal group and prioritization matrix determining how the target of the project is to improve the care for syphilis; analyzing the root causes of the problem based on a flowchart of care for gestational syphilis; constructing and validating criteria to evaluate quality; evaluating the level of quality, measuring the level of compliance with the criteria; planning and implementing the improvement intervention; and reassessing the level of quality to test the effect of the intervention.

The elaboration was participatory and involved an improvement team composed of the technical team of social organization (SO), administrators of the Coordination of Planning Area 3.3 (CPA), UBS administrators and professionals of the family health teams. It was based on data, with actions directed to the quality criteria of worst compliance in the first evaluation.

**Figure 1:**
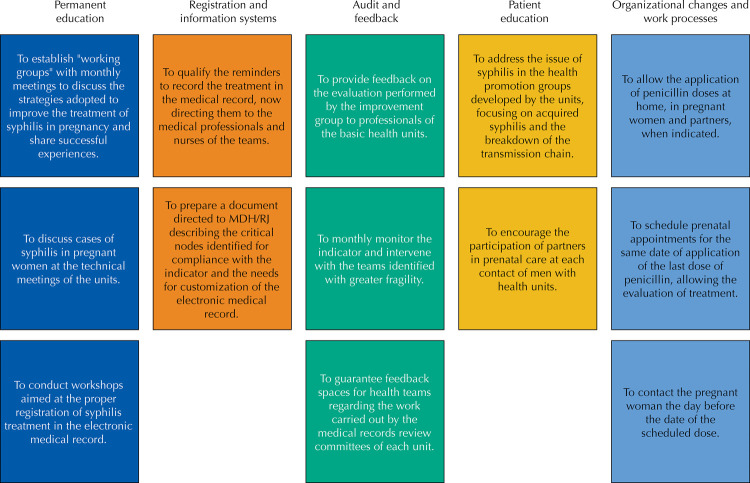
Diagram of affinities with the synthesis of the proposed interventions.

The intervention was multifaceted, and the various actions were grouped into related areas of an affinity diagram (Figure 1), with subgroups of interventions proposed by WHO to improve quality in health systems^18^. The implementation of the intervention followed a plan that specified its actions, people responsible for it and deadlines.

The first intervention was the presentation of the results of the evaluation to the SO and CPA administrators, then to the UBS administrators and health professionals. From then on, the other intervention proposals were collectively elaborated in face-to-face workshops with the members of the improvement team at all UBS. The agreement of the deadlines and the feasibility of the actions were periodically reviewed, in order to adjust to reality.

**Figure 2:**
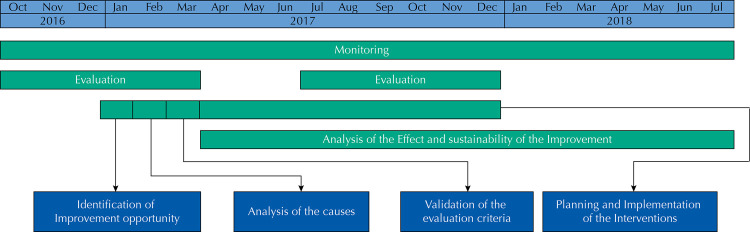
Timeline of quality improvement project.

All actions were initially proposed to take place continuously and with the active participation of the improvement team, but, due to the influence of contextual factors, which will be detailed below, the actions were mostly carried out by the UBS teams under the coordination of their administrators. Figure 2 shows the implementation schedule of the improvement project.

To analyze the effect of the intervention, we defined ten quality criteria of care for syphilis, based on national^19,20^ and international^21,22^ guidelines, namely: (1) performing the treponemal test (rapid syphilis test) in the first prenatal consultation; (2) requesting a non-treponemal test (*venereal disease research laboratory* – VDRL) when the treponemal is positive in the first prenatal consultation; (3) notifying the condition (to the *Sistema Nacional de Agravos de Notificação* – SINAN) for all pregnant women with positive rapid test or VDRL; (4) activating the International Classification of Diseases (ICD) of syphilis in the electronic medical record at the moment of diagnosis (positive rapid test or VDRL); (5) adequate treatment scheme according to protocol and technical note (adequate drug, dosage and interval); (6) testing the sexual partnership(s) of the pregnant women with positive syphilis; (7) concomitant treatment of the sexual partner(s) of the pregnant women with positive syphilis; (8) post-treatment monitoring of syphilis in pregnant women (control VDRL); (9) treatment concluded at least 30 days prior to delivery; and (10) proper register of the treatment of the pregnant women and her partner(s) in the electronic medical record (specific fields).

For monitoring purposes, the indicator of the proportion of syphilis notifications in pregnancy with adequate treatment was considered, which was already monitored by the improvement team as the goal of the management contract of the Municipal Health Department (MHD) with SO. The indicator defined as adequate treatment the appropriate prescription for the type of syphilis, the treatment of the partner and the completion of treatment at least 30 days before the date of delivery. Diagnostic coding of pregnancy and syphilis according to ICD, registration of the notification number in SINAN and rapid testing were also mandatory for accounting.

Quality assessments took place retrospectively, in two moments, before and after the intervention. We studied the selected criteria in all pregnant women who fit the characteristics of the study population (70 cases in the first evaluation and 108 cases in the second evaluation). In the case of the monitoring indicator, all cases that met the inclusion criterion were also evaluated, with an average of 127 pregnant women per month.

Data were collected from the medical records of pregnant women with reported gestational syphilis and prenatal care ended in the period under analysis. The first evaluation (pre-intervention) was made from October 2016 to March 2017, and the second from July to December 2017. To analyze the effect and sustainability of the improvement achieved, we monitored the indicator until July 2018.

Data analysis included point estimates of criteria compliance and, to estimate the improvement achieved, the estimation of absolute and relative improvements for each criterion. Statistical significance was analyzed with a unilateral hypothesis test for quality improvement, considering as null hypothesis the absence of improvement, which was rejected when the p-value was less than 0.05. In the analysis of the time series of the indicator, the statistical control rules were tested to identify trends in the evaluated process.

The contextual factors and their influence on the improvement results were considered by the improvement team from synthetic analysis based on the Model for Understanding Success in Quality (MUSIQ)^23^.

The project was submitted to the system of the Research Ethics Committee of the Comissão Nacional de Ética em Pesquisa (REC/CONEP) and approved under opinion No. 2.803.062.

## RESULTS

The initial evaluation revealed serious quality problems in gestational syphilis care. The criteria for reporting the disease of pregnant women with a positive test, activation of syphilis ICD in the electronic medical record at the time of diagnosis, post-treatment monitoring of syphilis in the pregnant woman, testing of sexual partnerships, treatment of sexual partnerships and adequate registration of the treatment of the pregnant woman in the medical record had the lowest adherence, with less than 65% compliance (Table).

**Table:** Compliance with the quality criteria of syphilis treatment in pregnancy before and after the intervention and improvement achieved.

Criterion	First evaluation (p1)*	Second evaluation (p2)*	Absolute improvement (p2−p1)	Relative improvement (p2−p1 / 100−p1)	Statistical significance (p)
1. Performing a treponemal test (rapid syphilis test) at the first prenatal appointment.	77.1	85.7	8.6	37.5	> 0.05
2. Non-treponemic test (VDRL) request when treponemal is positive at the first prenatal appointment.	74.3	70.4	-3.9	-15.2	-
3. Notifying about the disease (SINAN) for all pregnant women with a positive rapid test or VDRL.	62.8	78.5	15.7	42.2	0.018
4. Activation of syphilis ICD in the electronic medical record at the time of diagnosis (positive rapid test or VDRL).	62.8	71.4	8.6	23.1	> 0.05
5. Appropriate treatment scheme according to protocol and technical note (appropriate drug, dosage and interval).	91.4	99.1	7.7	89.5	0.004
6. Testing of sexual partnership(s) of pregnant women with positive syphilis.	42.8	48.5	5.7	9.9	> 0.05
7. Concomitant treatment of sexual partnership(s) of pregnant women with positive syphilis.	42.8	44.2	1.4	2.4	> 0.05
8. Post-treatment monitoring of syphilis in pregnant women (control VDRL).	61.4	51.4	-10	-25.9	-
9. Treatment completed at least 30 days before delivery.	85.7	98.5	12.8	89.5	< 0.001
10. Adequate record of the treatment of the pregnant woman and her partnership(s) in the electronic medical record (specific fields).	42.8	62.8	20	34.9	0.005
Composite indicator: quality of syphilis care during pregnancy.	64.4	71	6.7	28.8	> 0.05

* p1 and p2: percentage of compliance in the first and second evaluation, respectively. VDRL: venereal disease research laboratory; SINAN: *Sistema Nacional de Agravos De Notificação*; ICD: International Classification of Diseases.

**Figure 3:**
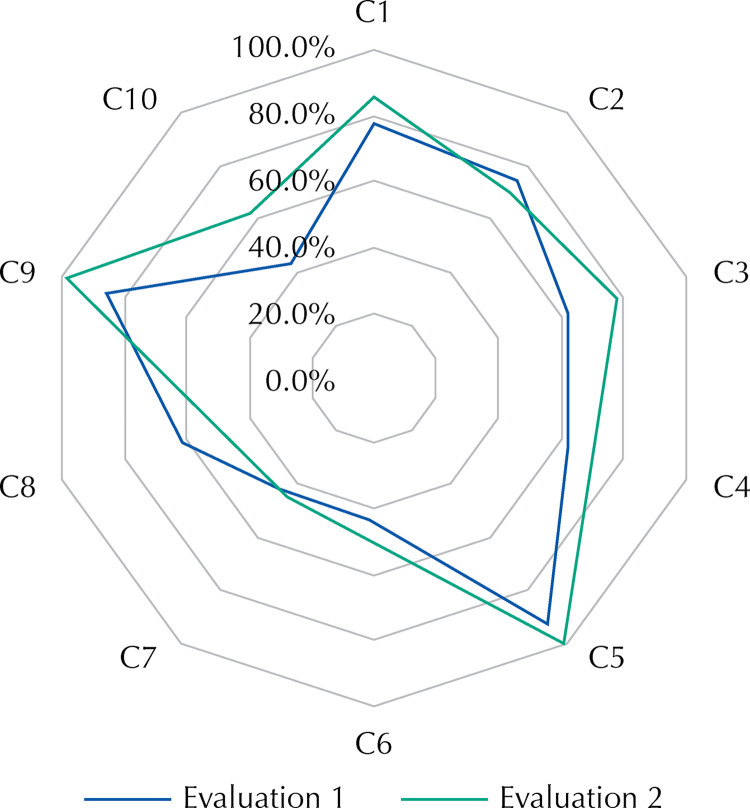
Radar chart with compliance with the quality criteria (C1–C10) of the treatment of syphilis in pregnancy before and after the improvement intervention.

Considering the ten criteria studied as components of a single indicator of “quality of care for gestational syphilis”, we observed an absolute total improvement of 6.7% (64.4% versus 71.0%) and relative improvement of 28.8% (p > 0.05) after the intervention. This was useful to improve five of the six syphilis care quality criteria that were prioritized after the first evaluation (Table; Figure 3). As for the four criteria that showed good levels of compliance in the first evaluation, three also improved.

Regarding the statistical significance of the improvement shown in the Table, four criteria obtained p-value less than 0.05, characterizing a statistically significant improvement in the quality level. They are: notification of the pregnant woman’s condition with a positive test; adequate treatment schedule; treatment completed at least 30 days before delivery; and adequate registration of the pregnant woman’s treatment and her partner(s) in the electronic medical record. On the other hand, it is evident that the criteria of testing and treating sexual partnerships were the ones that showed the least significant improvements.

Although they were not prioritized in the intervention, three criteria that in the first evaluation showed high levels of compliance also obtained improvement in the reassessment. The treatment schedule according to protocol and the completion of treatment at least 30 days before delivery showed significant improvement (p < 0.05).

**Figure 4:**
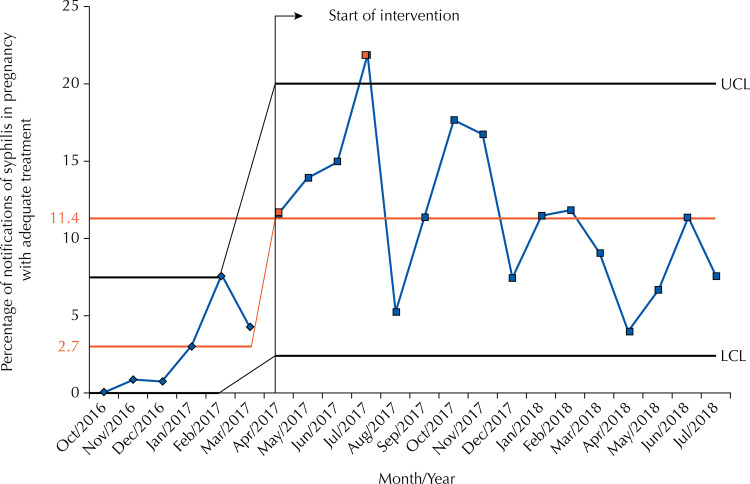
Statistical control chart with the performance of the indicator of monitoring adequate treatment of syphilis in pregnancy.

LCL: Lower control limit; UCL: Upper control limit.

In the monitoring of the administrator contract indicator (Figure 4) there was a significant improvement, especially from April 2017 — the initial period of the interventions —, which can be observed by the increase in the average percentage of 2.7%, before, to 11.4% after the intervention. The improvement obtained was significant, despite the fact that the performance remained low throughout the project and the increase in variation (standard deviation of 1.5 versus 3.0).

After reassessment, the quality level worsened in two criteria: the non-treponemal test request at the first prenatal appointment and the post-treatment monitoring, both related to the VDRL examination request (Table).

The implementation of the planned intervention had several obstacles to its effectiveness. However, the most consolidated interventions were: the evaluation feedback for professionals directly responsible for care; qualification workshops of registration in the electronic medical record; qualification of the appropriate registration reminders triggered for health professionals; and direct intervention of the improvement team in the teams identified with greater fragility in syphilis care.

Among the factors of MUSIQ^23^, the factors that most contributed to the success of the intervention were the external regulatory pressure to improve the performance of the contracted indicator, the leadership of the UBS administrators, the commitment and motivation of the health team professionals, the available data infrastructure and the interest of the SO to emphasize the improvement of quality as part of its strategic objectives.

On the other hand, the contextual factors that most hindered the success of the project and the stability of the results achieved were the governance crisis arising from the shared administration model between SO and CPA/MHD and, mainly, the political-economic crisis of the municipality, which had a great impact on the availability of financial and human resources.

## DISCUSSION

Coping with syphilis is an urgent need in Brazil and in several countries. Although there is evidence about the main failures in the care process^24,25^, there is a great lack of studies that show how to improve this public health problem. Published experiences of improving the quality of syphilis care are often limited specifically to the expansion of diagnosis^26,27^.

This study contributes to identify priorities throughout the *continuum* of gestational syphilis care in primary health care, showing a concise and practical evaluation model to analyze the quality level in the local context and design improvement interventions adjusted to their priorities. It also provides a model for conducting participatory projects or improvement cycles that stimulate the implementation of good practices.

The positive points of the study are the primary data collection from patients’ medical records — considered advantageous when compared with secondary collection via notification system —, the large number of health units and teams involved in the project and the improvement achieved, even in a challenging context for implementing and maintaining interventions.

The intervention was based on data and focused mainly on the most problematic criteria. Even so, eight of the ten criteria improved, indicating that indirect actions may have influenced virtually all aspects of the care line. This must have occurred mainly because of organizational changes and work processes.

Before the intervention, the level of quality of care for gestational syphilis was unsatisfactory, with weaknesses in the approach to sexual partnerships, therapeutic follow-up and registration of health information. However, after the intervention, improvement was observed in these main weaknesses, with emphasis on the recording of information. This evolution was associated with the interventions on “registration and information systems” and “permanent education”, which were the best implemented in the project.

On the other hand, there were difficulties in significantly improving the criteria related to sexual partnerships of pregnant women with syphilis. The approach of these partnerships in the treatment of sexually transmitted infections (STIs) is the subject of constant discussion and constitutes a great challenge throughout Brazil. Data from the Ministry of Health indicate that, in 2016, 62.2% of sexual partnerships of cases of syphilis in pregnant women reported in the country were not treated^2^.

Still, in 2017, the Ministry of Health published an informative note^28^ that changed the criteria for defining cases for reporting syphilis, so that, for epidemiological surveillance purposes, the treatment of the sexual partnership of the mother is no longer considered as a criterion for defining cases of congenital syphilis. This modification, if not well understood by health professionals, can be confused with a care criterion and generate the false impression that there is no need for treating sexual partnerships, both in cases of syphilis and other STIs.

The great social vulnerability in the Planning Area 3.3 made the treatment of sexual partnerships especially challenging, given the migration characteristics of residents, the multiplicity of sexual partnerships and the high rate of urban violence in the area, related to drug trafficking — relatively often, health professionals are faced with partners kept in the prison system and with worse access to treatment.

Two criteria showed worse compliance in the reassessment: non-treponemal testing at the first appointment and during monitoring. Diagnostic confirmation with non-treponemal tests, such as VDRL, is essential to differentiate active disease from serological scar; and the monitoring conducted from these tests is essential to classify the response to treatment and to define the most correct conduct for each case^9^. Regarding monitoring, another intervention project to improve care for gestational syphilis suggests that patient education is essential, since the main cause of the problem of therapeutic follow-up may be the lack of consultations, so that patient reminder systems are suggested^29^. In addition, there are reports that behavioral interventions have contributed to the effectiveness of improving the care of pregnant women with syphilis in African countries^30^.

We had difficulties in performing some of the proposed actions due to intrinsic characteristics of the project. As an initiative outside the UBS, one of the biggest challenges was to involve front-line health professionals in the changes. Thus, the project improvement team limited itself to inducing the improvement processes, but there was great variation in the adherence of the 26 target units. This reinforces the need for participation and involvement of internal actors, because, according to Palmer^31^ “from the outside we can evaluate, but only from the inside we can evaluate and improve.”

Another explanation for the lack of effect for the VDRL-related criteria was the difficulty in accessing the exams. In June 2017, MDH/RJ broke a contract with the clinical analysis laboratory that performed the VDRL tests, and then hired two laboratories that had numerous problems, from the shortage of inputs to the delay or non-delivery of test results.

Valuing the role of context in the scope of the science of improving health care stems from the consensus that interventions in this sense do not develop in sterile or laboratory spaces. Factors facilitate or hinder the implementation of the intervention, influencing its effectiveness and sustainability^13^, which makes the context an important modulator of the effect of quality improvement interventions. MUSIQ suggests that we should deepen the understanding of mechanisms by which the context interferes with the outcomes of health interventions^23^.

The external pressure to improve the performance of the monitoring indicator of contracted syphilis was considered an important contextual factor, having contributed positively to the success of the intervention. Since the end of the contract was approaching, and achieving targets of the indicators was one of the main requirements for its renewal, improvement actions became part of the strategic objectives of SO.

Another relevant factor was the commitment to the leadership development of UBS administrators as a strategic element for improvement^23^. Administrators have, among others, the duty of mediating conflicts, managing productive processes in the health field and managing work with a focus on the quality of patient service^32^. Thus, and due to their high capillarity and to the leadership capacity of health professionals, they contributed to the receptivity and involvement of family health teams^23^.

On the other hand, the governance crisis arising from the shared administration model between SO and CPA/MHD, characterized by the lack of clarity of the roles of the contractual parties, hindered the implementation of several actions. Apart from this, and even more forcefully, the political-economic crisis experienced by the municipality from 2017 has generated a great impact on the availability of financial and human resources for the execution of interventions, in addition to aggravating the issue of the governance crisis.

Caution is necessary when generalizing the results of the evaluation of quality of this study to other Brazilian regions. For example, the high levels of compliance in the criteria of performing treponemal testing and adequate treatment scheme may not be the reality for most Brazilian municipalities. Data from the *Programa Nacional de Melhoria do Acesso e da Qualidade da Atenção Básica* (PMAQ-AB – National Program for Improving Access and Quality of Primary Care), obtained in 2012 and 2014, showed that the availability of rapid tests for syphilis and penicillin benzathine was low throughout the country^33^.

Regarding the effectiveness of the implemented intervention, when considering its design without a control group, it was not possible to rule out that other concomitant initiatives of induction and quality improvement may have influenced the results obtained. Although this is a limitation, we consider that it is very difficult to conduct this type of study in a controlled way, since a great logistics is necessary to select, evaluate and involve many health services.

In addition, the study did not use any outcome indicators to measure the level of quality, such as syphilis cure, neonatal morbidity and mortality, costs etc. It was not possible to access these data on the specific population of the study; however, we understand that there was no prejudice to its greater purpose of increasing adherence to good evidence-based practices and analyzing the impact of the project on improving the indicator of adequate treatment for gestational syphilis.

## CONCLUSIONS

The initial assessment of the quality of care for gestational syphilis in primary health care identified improvement priorities that were met with relative success, despite contextual factors.

The observed effect was mainly attributed to the actions of permanent education, registration and information systems, and audit and feedback. Moreover, the actions of organizational changes and work processes, even if implemented at different levels in each UBS, were also considered essential for the improvement results achieved.

The improvement cycle was considered adequate to implement changes and encourage good practices, and should be continued to achieve an acceptable level of quality for all criteria, including those that, despite having achieved significant improvements, still represent important public health problems, such as underreporting. The problems identified, as well as the contextual modulators of the effect, should be considered in future interventions.
