# Supplementary material for: Improvement of quality of care for gestational syphilis in the municipality of Rio de Janeiro
Source: Rev Saude Publica. 2021 Jun 7;55:34. doi: 10.11606/s1518-8787.2021055002534 (PMC8225322; doi:10.11606/s1518-8787.2021055002534)
Supplement: Translation

# Translation: Melhoria da qualidade do cuidado à sífilis gestacional no município do Rio de Janeiro

**Authors:** Brena Gabriella Tostes de Cerqueira, Eliane Pereira da Silva, Zenewton André da Silva Gama

**Affiliations:** I Brasil Universidade Federal do Rio Grande do Norte. Centro de Ciências da Saúde. Programa de Pós-Graduação em Gestão da Qualidade em Serviços de Saúde. Natal, RN, Brasil; II Brasil Universidade Federal do Rio Grande do Norte. Centro de Ciências da Saúde. Departamento de Medicina Clínica. Natal, RN, Brasil; III Brasil Universidade Federal do Rio Grande do Norte. Centro de Ciências da Saúde. Departamento de Saúde Coletiva. Natal, RN, Brasil

**Keywords:** Sífilis, Gravidez, Melhoria de Qualidade, Qualidade da Assistência à Saúde, Atenção Primária à Saúde

## Abstract

**OBJETIVO:**

Analisar o efeito de uma intervenção multifacetada no cuidado das gestantes com sífilis na atenção primária à saúde.

**MÉTODOS:**

Trata-se de projeto de melhoria da qualidade realizado em 26 unidades básicas de saúde do município do Rio de Janeiro, entre janeiro e dezembro de 2017. O desenho foi quase-experimental misto, com análises anteriores, posteriores e de série temporal. Avaliou-se o cuidado prestado a todas as gestantes com sífilis e pré-natal encerrado no período, mediante dez critérios de qualidade e um indicador. A intervenção foi multifacetada, abrangendo educação permanente, melhoria dos registros e sistemas de informação, auditoria e *feedback*, educação do paciente, mudanças organizacionais e nos processos de trabalho. Estimaram-se as melhorias absoluta e relativa dos critérios e sua significância estatística (α = 5%). Os facilitadores e dificultadores da intervenção foram analisados segundo o *Model for Understanding Success in Quality*.

**RESULTADOS:**

Após a intervenção, observou-se melhoria absoluta total de 6,7% (64,4% *versus* 71,0%) e relativa de 28,8% (p > 0,05). Oito dos dez critérios de qualidade tiveram melhoria, sendo esta significativa em quatro deles (p < 0,05). O indicador mensal de tratamento adequado também melhorou (p < 0,05), porém manteve baixo desempenho em todo o projeto. Destacou-se positivamente o aumento da conformidade do esquema de tratamento com o protocolo (91,4% *versus* 99,1%), porém as principais oportunidades de melhoria foram a testagem (42,8% *versus* 48,5%) e o tratamento das parcerias sexuais (42,8% *versus* 44,2%). Pressões regulatórias para melhorar o indicador mensal e a crise político-econômica vivenciada pelo município modularam o efeito da intervenção.

**CONCLUSÃO:**

O projeto foi útil para identificar prioridades e orientar intervenções para a melhoria da qualidade da assistência à sífilis, embora ainda exista ampla margem para avanços. Os problemas identificados, bem como os moduladores contextuais do efeito, devem ser considerados em futuras intervenções.

## INTRODUÇÃO

O aumento da incidência de sífilis em diversos países do mundo tem sido tratado como um grave problema de saúde pública. Esse cenário epidemiológico é uma realidade no Brasil^1^, pois as taxas de incidência de sífilis congênita e gestacional quase triplicaram entre 2010 e 2016, passando de 2,4 para 6,8 e de 3,5 para 12,4 casos por mil nascidos vivos, respectivamente^2^. Dados mais recentes do Ministério da Saúde indicam que as mesmas taxas em 2018 já eram de 9,0 e 21,4, persistindo a tendência de crescimento^3^.

Prevenir a transmissão vertical da sífilis é uma prioridade do Ministério da Saúde brasileiro, alinhada com a Organização Pan-Americana da Saúde (Opas)^4^. Em 2016, um relatório da Opas^5^ contendo dados de países e territórios da América Latina e Caribe apontou a necessidade de reforçar as ações estratégicas e o plano de ação começados em 2010 para eliminar a transmissão vertical do HIV e da sífilis, uma vez que as metas estabelecidas para 2015 ainda não haviam sido alcançadas por todos, sendo elas: redução da taxa de transmissão vertical e de incidência do HIV (≤ 2% e até 0,3 caso por mil nascidos vivos, respectivamente); redução da taxa de incidência de sífilis congênita (0,5 caso por mil nascidos vivos); ampliação da cobertura de pré-natal com pelo menos uma consulta (≥ 95%); ampliação da cobertura de testagem de HIV e sífilis em gestantes (≥ 95%); ampliação da cobertura de tratamento com antirretrovirais em gestantes soropositivas (≥ 95%); e ampliação da cobertura de tratamento com penicilina em gestantes com sífilis (≥ 95%)^6^.

Uma iniciativa nacional que baliza o enfrentamento à sífilis é a Agenda de Ações Estratégicas para Redução da Sífilis Congênita no Brasil^4^, lançada em 2016, que estabelece prioridades para a qualificação da atenção à saúde e o compartilhamento de responsabilidades. Além disso, em 2017 destaca-se o Projeto de Resposta Rápida à Sífilis nas Redes de Atenção, que tem como objetivo reduzir a sífilis adquirida em gestantes e eliminar a sífilis congênita no Brasil mediante o fortalecimento e desenvolvimento de ações estratégicas de abrangência universal e de ações com municípios considerados prioritários, selecionados por critérios epidemiológicos^7^.

Estudos apontam que início tardio do pré-natal, baixo número de consultas, falta ou demora no diagnóstico, esquema terapêutico inadequado ou incompleto, não tratamento das parcerias sexuais e falta de conhecimento da gestante sobre a doença são as principais falhas relacionadas ao manejo inadequado da sífilis gestacional^8^. O diagnóstico precoce e o tratamento oportuno e adequado das gestantes e parcerias sexuais resultam na redução da morbimortalidade associada à transmissão vertical e são considerados marcadores da qualidade da assistência pré-natal^4^.

Em 2018, dentre os estados brasileiros, o Rio de Janeiro destacou-se com as maiores taxas de sífilis gestacional e congênita, concentrando 28,4% de todos os óbitos por sífilis congênita em menores de um ano de idade no Brasil^3^. Dentre as capitais, a cidade do Rio de Janeiro tinha a segunda maior taxa de detecção de sífilis em gestantes do país, com 51,5 casos por mil nascidos vivos, e uma taxa de incidência de sífilis congênita de 13,4 – maior do que a média nacional, de 9,0, e bem acima da meta estabelecida pela Organização Mundial da Saúde (OMS)^3^, de menos de 0,5 caso por mil nascidos vivos.

Enfrentar essa questão é um grande desafio para os municípios brasileiros, mas a utilização de métodos e ferramentas de gestão da qualidade pode ser fundamental para uma resposta organizada ao problema. Dentre os processos de gestão da qualidade (planejamento da qualidade, monitoramento da qualidade e ciclos de melhoria)^11^, os ciclos de melhoria possibilitam a obtenção de resultados para problemas complexos em prazo relativamente curto, permitindo avaliar o problema e testar ciclicamente mudanças que podem ser monitoradas através do controle estatístico^14^.

Diante do exposto, este estudo buscou avaliar e melhorar a qualidade da assistência à sífilis gestacional, no âmbito da atenção primária à saúde, em uma das áreas de maior incidência de sífilis no Brasil. Especificamente, o projeto objetivou: (1) avaliar a qualidade da atenção prestada às gestantes diagnosticadas com sífilis a partir da mensuração de critérios e indicadores de qualidade; e (2) analisar o efeito de uma intervenção multifacetada para melhorar a qualidade do cuidado.

## MÉTODOS

Trata-se de um projeto de melhoria da qualidade com desenho de estudo quase-experimental, sem grupo controle e misto, combinando análise de série temporal e avaliações anteriores e posteriores^15^. Foi desenvolvido no município do Rio de Janeiro, nas unidades básicas de saúde (UBS) da Área de Planejamento 3.3, que abarca o segundo maior contingente populacional do município, está localizada na Zona Norte da cidade e aglutina 29 bairros – muitos deles com os menores índices de desenvolvimento humano (IDH) e maiores índices de violência urbana do município^16,17^. Durante o desenvolvimento do estudo, essa área possuía 31 UBS e 180 equipes de saúde da família, o que correspondia a aproximadamente 70% de cobertura pelo modelo da Estratégia Saúde da Família (ESF).

Para fins de definição da amostragem, foram excluídas as unidades que não utilizavam prontuário eletrônico ou que haviam sido inauguradas nos últimos seis meses. Sendo assim, 26 UBS foram incluídas no estudo (154 equipes de saúde da família).

A intervenção de melhoria foi elaborada seguindo um modelo de ciclo de melhoria da qualidade^11^. Os passos do projeto de melhoria foram: identificação e priorização da oportunidade de melhoria de forma participativa e baseada em critérios, utilizando técnica do grupo nominal e matriz de priorização, elegendo como alvo do projeto a melhoria da assistência à sífilis; análise das causas do problema com base em um fluxograma da assistência à sífilis gestacional; construção e validação de critérios de avaliação da qualidade; avaliação do nível de qualidade, com medição do nível de cumprimento dos critérios; planejamento e implementação da intervenção de melhoria; e reavaliação do nível de qualidade para testar o efeito da intervenção.

A elaboração foi participativa e envolveu uma equipe de melhoria composta pela equipe técnica da organização social (OS), por gestores da Coordenação da Área de Planejamento 3.3 (CAP), pelos gerentes das UBS e por profissionais das equipes de saúde da família. Foi baseada em dados, com ações direcionadas aos critérios de qualidade de pior conformidade na primeira avaliação.

A intervenção foi multifacetada, sendo as diversas ações agrupadas em áreas afins de um diagrama de afinidades (Figura 1), com subgrupos de intervenções propostas pela OMS para melhoria da qualidade em sistemas de saúde^18^. A implementação da intervenção seguiu um plano que especificava suas ações, seus responsáveis e prazos.

**Figura 1:** Diagrama de afinidades com a síntese das intervenções propostas.

A primeira intervenção feita foi a apresentação dos resultados da avaliação aos gestores da OS e da CAP e, em seguida, aos gerentes das UBS e aos profissionais de saúde. A partir de então, as demais propostas de intervenção foram coletivamente elaboradas em oficinas presenciais com os membros da equipe de melhoria em todas as UBS. A pactuação dos prazos e a viabilidade das ações foram revisadas periodicamente, de forma a se ajustarem à realidade.

Todas as ações foram inicialmente propostas para acontecerem de forma contínua e com participação ativa da equipe de melhoria, mas, por influência de fatores contextuais, que serão detalhados adiante, as ações foram, em sua maioria, realizadas pelas equipes das UBS sob coordenação de seus gerentes. O cronograma de execução do projeto de melhoria foi ilustrado na Figura 2.

**Figura 2:** Linha do tempo do projeto de melhoria da qualidade.

Para analisar o efeito da intervenção, foram definidos dez critérios de qualidade da assistência à sífilis, baseados em diretrizes nacionais^19,20^e internacionais^21,22^, a saber: (1) realização de teste treponêmico (teste rápido de sífilis) na primeira consulta de pré-natal; (2) solicitação de teste não treponêmico (*venereal disease research laboratory* – VDRL) quando houver treponêmico positivo na primeira consulta de pré-natal; (3) realização da notificação do agravo (no Sistema Nacional de Agravos de Notificação – Sinan) para todas as gestantes com teste rápido ou VDRL positivo; (4) ativação da Classificação Estatística Internacional de Doenças e Problemas Relacionados com a Saúde (CID) de sífilis no prontuário eletrônico no momento do diagnóstico (teste rápido ou VDRL positivo); (5) esquema de tratamento adequado conforme protocolo e nota técnica (droga, dosagem e intervalo adequados); (6) testagem da(s) parceria(s) sexual(s) de gestantes positivas para sífilis; (7) tratamento concomitante da(s) parceria(s) sexual(s) de gestantes positivas para sífilis; (8) monitoramento pós-tratamento de sífilis na gestante (VDRL de controle); (9) tratamento concluído pelo menos 30 dias antes do parto; e (10) registro adequado do tratamento da gestante e de sua(s) parceria(s) no prontuário eletrônico (campos específicos).

Para fins de monitoramento, foi considerado o indicador de proporção de notificações de sífilis na gestação com tratamento adequado, que já era acompanhado pela equipe de melhoria como meta do contrato de gestão da Secretaria Municipal de Saúde (SMS) com a OS. O indicador definia como tratamento adequado a prescrição apropriada para o tipo de sífilis, o tratamento do parceiro e a conclusão do tratamento pelo menos 30 dias antes da data do parto. Eram obrigatórios também, para a contabilização, a codificação diagnóstica da gestação e da sífilis conforme a CID, o registro do número da notificação no Sinan e a realização do teste rápido.

As avaliações da qualidade aconteceram retrospectivamente, em dois momentos, antes e após a intervenção. Os critérios selecionados foram pesquisados em todas as gestantes que se enquadravam nas características da população do estudo (70 casos na primeira avaliação e 108 casos na segunda avaliação). No caso do indicador de monitoramento, foram avaliados também todos os casos que cumpriam o critério de inclusão, com média de 127 gestantes por mês.

Os dados foram colhidos dos prontuários de gestantes com sífilis gestacional notificada e pré-natal encerrado no período em análise. A primeira avaliação (pré-intervenção) foi feita de outubro de 2016 a março de 2017, e a segunda avaliação de julho a dezembro de 2017. Para análise do efeito e da sustentabilidade da melhoria alcançada, o monitoramento do indicador seguiu até julho de 2018.

A análise dos dados incluiu as estimativas pontuais de conformidade dos critérios e, para estimar a melhoria alcançada, o cálculo das melhorias absoluta e relativa de cada critério. A análise da significância estatística foi feita com teste de hipótese unilateral para a melhoria da qualidade, considerando como hipótese nula a ausência de melhoria, que se rejeitava quando o p-valor era inferior a 0,05. Na análise da série temporal do indicador, foram testadas as regras de controle estatístico para identificar tendência no processo avaliado.

Os fatores contextuais e sua influência sobre os resultados de melhoria foram considerados pela equipe de melhoria a partir de análise sintética baseada no *Model for Understanding Success in Quality* (Musiq)^23^.

O projeto foi submetido ao sistema do Comitê de Ética em Pesquisa da Comissão Nacional de Ética em Pesquisa (CEP/Conep) e aprovado sob o parecer nº 2.803.062.

## RESULTADOS

A avaliação inicial revelou graves problemas de qualidade na assistência à sífilis gestacional. Os critérios de notificação do agravo das gestantes com teste positivo, ativação do CID de sífilis no prontuário eletrônico no momento do diagnóstico, monitoramento pós-tratamento de sífilis na gestante, testagem de parcerias sexuais, tratamento das parcerias sexuais e registro adequado do tratamento da gestante no prontuário foram os de menor adesão, com menos de 65% de cumprimento (Tabela).

**Tabela:** Cumprimento dos critérios de qualidade do tratamento de sífilis na gestação antes e depois da intervenção e melhoria alcançada.

Critério	Primeira avaliação (p1)*	Segunda avaliação (p2)*	Melhoria absoluta (p2−p1)	Melhoria relativa (p2−p1 / 100−p1)	Significância estatística (p)
1. Realização de teste treponêmico (teste rápido de sífilis) na primeira consulta de pré-natal.	77,1	85,7	8,6	37,5	> 0,05
2. Solicitação de teste não treponêmico (VDRL) quando treponêmico positivo na primeira consulta de pré-natal.	74,3	70,4	-3,9	-15,2	-
3. Realização da notificação do agravo (Sinan) para todas as gestantes com teste rápido ou VDRL positivo.	62,8	78,5	15,7	42,2	0,018
4. Ativação do CID de sífilis no prontuário eletrônico no momento do diagnóstico (teste rápido ou VDRL positivo).	62,8	71,4	8,6	23,1	> 0,05
5. Esquema de tratamento adequado conforme protocolo e nota técnica (droga, dosagem e intervalo adequados).	91,4	99,1	7,7	89,5	0,004
6. Testagem da(s) parceria(s) sexual(s) de gestantes positivas para sífilis.	42,8	48,5	5,7	9,9	> 0,05
7. Tratamento concomitante da(s) parceria(s) sexual(s) de gestantes positivas para sífilis.	42,8	44,2	1,4	2,4	> 0,05
8. Monitoramento pós-tratamento de sífilis na gestante (VDRL de controle).	61,4	51,4	-10	-25,9	-
9. Tratamento concluído pelo menos 30 dias antes do parto.	85,7	98,5	12,8	89,5	< 0,001
10. Registro adequado do tratamento da gestante e de sua(s) parceria(s) no prontuário eletrônico (campos específicos).	42,8	62,8	20	34,9	0,005
Indicador composto: qualidade da assistência à sífilis na gestação.	64,4	71	6,7	28,8	> 0,05

* p1 e p2: porcentagem de conformidade na primeira avaliação e na segunda avaliação, respectivamente. VDRL: *venereal disease research laboratory*; Sinan: Sistema Nacional de Agravos de Notificação; CID: Classificação Estatística Internacional de Doenças e Problemas Relacionados com a Saúde.

Considerando os dez critérios pesquisados como componentes de um único indicador de “qualidade da assistência à sífilis gestacional”, observou-se melhoria absoluta total de 6,7% (64,4% *versus* 71,0%) e relativa de 28,8% (p > 0,05) após a intervenção. Esta foi útil para melhorar cinco dos seis critérios de qualidade da atenção à sífilis que foram priorizados após a primeira avaliação (Tabela; Figura 3). Quanto aos quatro critérios que apresentavam bons níveis de cumprimento na primeira avaliação, três também tiveram melhoria.

**Figura 3:** Gráfico de radar com o cumprimento dos critérios de qualidade (C1–C10) do tratamento de sífilis na gestação antes e depois da intervenção de melhoria.

Em relação à significância estatística da melhoria apresentada na Tabela, quatro critérios obtiveram p-valor menor que 0,05, caracterizando uma melhoria estatisticamente significativa do nível de qualidade. São eles: realização da notificação do agravo das gestantes com teste positivo; esquema de tratamento adequado; tratamento concluído pelo menos 30 dias antes do parto; e registro adequado do tratamento da gestante e de sua parceria no prontuário eletrônico. Por outro lado, fica evidente que os critérios de testagem e tratamento das parcerias sexuais foram os que apresentaram melhorias menos expressivas.

Apesar de não terem sido priorizados na intervenção, três critérios que na primeira avaliação apresentaram níveis altos de cumprimento também obtiveram melhoria na reavaliação. O esquema de tratamento conforme protocolo e a conclusão do tratamento no mínimo 30 dias antes do parto apresentaram melhoria significativa (p < 0,05).

No monitoramento do indicador do contrato de gestão (Figura 4) houve melhoria relevante, especialmente a partir do mês de abril de 2017 – período inicial das intervenções –, o que pode ser observado pelo aumento da porcentagem média de 2,7%, antes, para 11,4% depois da intervenção. A melhoria obtida foi significativa, apesar de o desempenho se manter baixo em todo o projeto e do aumento da variação (desvio-padrão de 1,5 *versus* 3,0).

**Figura 4:** Gráfico de controle estatístico com o desempenho do indicador de monitoramento de tratamento adequado de sífilis na gestação.

LCI: Limite de controle inferior; LCS: Limite de controle superior.

Após reavaliação, o nível de qualidade piorou em dois critérios: a solicitação de teste não treponêmico na primeira consulta de pré-natal e o monitoramento pós-tratamento, ambos relacionados à solicitação de exame VDRL (Tabela).

A implementação da intervenção planejada teve diversos obstáculos à sua efetivação. No entanto, as intervenções mais consolidadas foram: o *feedback* da avaliação para os profissionais diretamente responsáveis pelo cuidado; as oficinas de qualificação do registro no prontuário eletrônico; a qualificação dos lembretes de registro adequado disparados para os profissionais de saúde; e a intervenção direta da equipe de melhoria nas equipes identificadas com maior fragilidade na assistência à sífilis.

Dentre os fatores do MUSIQ^23^, os que mais contribuíram para o sucesso da intervenção foram a pressão regulatória externa para melhorar o desempenho do indicador contratualizado, a liderança dos gerentes das UBS, o compromisso e a motivação dos profissionais das equipes de saúde, a infraestrutura de dados disponível e o interesse da OS em enfatizar a melhoria da qualidade como parte de seus objetivos estratégicos.

Por outro lado, os fatores contextuais que mais dificultaram o êxito do projeto e a estabilidade dos resultados alcançados foram a crise de governança oriunda do modelo de gestão compartilhada entre OS e CAP/SMS e, principalmente, a crise político-econômica do município, que teve grande impacto na disponibilidade de recursos financeiros e humanos.

## DISCUSSÃO

O enfrentamento à sífilis é uma necessidade urgente no Brasil e em diversos países. Apesar de existirem evidências sobre as principais falhas no processo de cuidado^24,25^, há grande carência de estudos que apontem como melhorar esse problema de saúde pública. As experiências publicadas de melhoria da qualidade da assistência à sífilis costumam se limitar especificamente à ampliação do diagnóstico^26,27^.

Este estudo contribuiu para identificar prioridades em todo o *continuum* do cuidado da sífilis gestacional na atenção primária à saúde, apresentando um modelo de avaliação conciso e prático para analisar o nível de qualidade no contexto local e desenhar intervenções de melhoria ajustadas às suas prioridades. Fornece também um modelo para a realização de projetos ou ciclos de melhoria participativos que estimulem a implementação de boas práticas.

Destacam-se como pontos positivos do estudo a coleta primária de dados nos prontuários das pacientes – considerada vantajosa quando comparada à coleta secundária via sistema de notificação –, o grande número de unidades e equipes de saúde envolvidas no projeto e a melhoria alcançada, mesmo em um contexto desafiador para implementação e manutenção das intervenções.

A intervenção foi baseada em dados e direcionada principalmente para os critérios mais problemáticos. Mesmo assim, oito dos dez critérios tiveram melhoria, o que indica que ações indiretas podem ter influenciado praticamente todos os aspectos da linha de cuidado. Isto deve ter ocorrido principalmente por causa das mudanças organizacionais e dos processos de trabalho.

Antes da intervenção, o nível de qualidade da assistência à sífilis gestacional mostrou-se insatisfatório, com fragilidades na abordagem das parcerias sexuais, no seguimento terapêutico e no registro da informação de saúde. No entanto, após a intervenção, observou-se melhoria nessas principais fragilidades, com destaque para o registro das informações. Tal evolução foi associada às intervenções sobre “Registro e sistemas de informação” e “Educação permanente”, que foram as mais bem implementadas no projeto.

Em contrapartida, houve dificuldades para melhorar de forma significativa os critérios relacionados às parcerias sexuais de gestantes com sífilis. A abordagem dessas parcerias no tratamento das infecções sexualmente transmissíveis (IST) é tema de constante discussão e configura um grande desafio em todo o Brasil. Dados do Ministério da Saúde indicam que, em 2016, 62,2% das parcerias sexuais dos casos de sífilis em gestantes notificados no país não foram tratadas^2^.

Ainda assim, em 2017 o Ministério da Saúde publicou uma nota informativa^28^ que alterou os critérios de definição de casos para notificação de sífilis, de forma que, para fins de vigilância epidemiológica, não se considera mais o tratamento da parceria sexual da mãe como critério de definição de caso de sífilis congênita. Essa modificação, se não bem entendida pelos profissionais de saúde, pode ser confundida com um critério assistencial e gerar a falsa impressão de não haver necessidade de tratamento das parcerias sexuais, tanto nos casos de sífilis quanto de outras IST.

A grande vulnerabilidade social na Área de Planejamento 3.3 tornou o tratamento das parcerias sexuais especialmente desafiador, dada a característica de migração dos moradores, a multiplicidade de parcerias sexuais e o alto índice de violência urbana na área, relacionada ao tráfico de drogas – com relativa frequência, os profissionais de saúde se deparam com parceiros reclusos no sistema prisional e com pior acesso ao tratamento.

Dois critérios apresentaram pior conformidade na reavaliação: a realização de teste não treponêmico na primeira consulta e no monitoramento. A confirmação diagnóstica com testes não treponêmicos, como o VDRL, é essencial para diferenciar doença ativa de cicatriz sorológica, e o monitoramento realizado a partir desses testes é fundamental para classificar a resposta ao tratamento e definir a conduta mais correta para cada caso^9^. Sobre o monitoramento, outro projeto de intervenção para melhoria da atenção à sífilis gestacional sugere que a educação do paciente é essencial, pois a principal causa do problema do seguimento terapêutico pode ser a falta às consultas, sugerindo-se, inclusive, sistemas de lembretes aos pacientes^29^. Além disso, há relatos de que intervenções comportamentais contribuíram para a efetividade da melhoria do cuidado de gestantes com sífilis em países da África^30^.

Tivemos dificuldades para realizar algumas das ações propostas devido a características intrínsecas do projeto. Por ser uma iniciativa externa às UBS, um dos maiores desafios foi envolver nas mudanças os profissionais de saúde da ponta. Assim, a equipe de melhoria do projeto limitou-se a induzir os processos de melhoria, mas houve grande variação na adesão das 26 unidades-alvo. Isso reforça a necessidade de participação e envolvimento dos atores internos, pois, segundo Palmer^31^, “de fora podemos avaliar, mas só de dentro podemos avaliar e melhorar”.

Outra explicação da ausência de efeito para os critérios relacionados ao VDRL foi a dificuldade de acesso aos exames. Em junho de 2017, a SMS/RJ rompeu contrato com o laboratório de análises clínicas que executava os exames de VDRL, sendo então contratados dois laboratórios que apresentaram inúmeros problemas, desde o desabastecimento de insumos até a demora ou não entrega de resultados dos exames.

A valorização do papel do contexto no âmbito da ciência da melhoria do cuidado de saúde decorre do consenso de que intervenções nesse sentido não se desenvolvem em espaços estéreis ou laboratoriais. Fatores facilitam ou dificultam a implementação da intervenção, influenciando sua efetividade e sustentabilidade^13^, o que faz do contexto um importante modulador do efeito das intervenções de melhoria da qualidade. O Musiq sugere que devemos aprofundar a compreensão dos mecanismos pelos quais o contexto interfere nos resultados das intervenções em saúde^23^.

A pressão externa para melhorar o desempenho do indicador de monitoramento da sífilis contratualizado foi considerada um fator contextual importante, tendo contribuído positivamente para o sucesso da intervenção. Visto que se aproximava o término do contrato e que o alcance das metas dos indicadores era um dos principais requisitos para sua renovação, as ações de melhoria se tornaram parte dos objetivos estratégicos da OS.

Outro fator relevante foi a aposta no desenvolvimento de liderança dos gerentes das UBS como elemento estratégico para a melhoria^23^. Os gerentes possuem, entre outras, as atribuições de mediar conflitos, gerenciar processos produtivos no campo da saúde e gerenciar o trabalho com foco na qualidade do atendimento ao usuário^32^. Assim, e por possuírem alta capilaridade e capacidade de liderança dos profissionais de saúde, eles contribuíram para a receptividade e o envolvimento das equipes de saúde da família^23^.

Por outro lado, a crise de governança oriunda do modelo de gestão compartilhada entre OS e CAP/SMS, caracterizada pela falta de clareza dos papéis das partes contratuais, dificultou a implementação de diversas ações. Afora isso, e de forma ainda mais contundente, a crise político-econômica vivenciada pelo município a partir de 2017 gerou grande impacto na disponibilidade de recursos financeiros e humanos para execução das intervenções, além de agravar a questão da crise de governança.

É necessária precaução ao generalizar para outras regiões brasileiras os resultados da avaliação da qualidade deste estudo. Por exemplo, os altos níveis de conformidade nos critérios de realização de teste treponêmico e de esquema de tratamento adequado podem não ser a realidade da maior parte dos municípios brasileiros. Dados do Programa Nacional de Melhoria do Acesso e da Qualidade da Atenção Básica (PMAQ-AB), obtidos em 2012 e 2014, mostraram que a disponibilidade de testes rápidos de sífilis e de penicilina benzatina era baixa em todo o país^33^.

Quanto à efetividade da intervenção implementada, considerando seu desenho sem grupo de controle, não foi possível descartar que outras iniciativas concomitantes de indução e melhoria da qualidade possam ter influenciado os resultados obtidos. Embora isso seja uma limitação, consideramos que é muito difícil realizar esse tipo de estudo de forma controlada, pois é necessária uma grande logística para selecionar, avaliar e envolver muitos serviços de saúde.

Além disso, o estudo não utilizou nenhum indicador de resultado para mensurar o nível de qualidade, tais como cura de sífilis, morbimortalidade neonatal, custos etc. Não foi possível acessar esses dados da população específica do estudo; contudo, entendemos que não houve prejuízo à sua finalidade maior de aumentar a adesão às boas práticas baseadas em evidências e de analisar o impacto do projeto na melhoria do indicador de tratamento adequado de sífilis gestacional.

## CONCLUSÕES

A avaliação inicial da qualidade da assistência à sífilis gestacional na atenção primária à saúde identificou prioridades de melhoria que foram enfrentadas com relativo sucesso, apesar dos fatores contextuais.

O efeito observado foi atribuído principalmente às ações de educação permanente, registro e sistemas de informação, e auditoria e *feedback*. Ademais, as ações de mudanças organizacionais e de processos de trabalho, mesmo que implementadas em níveis diferentes em cada UBS, também foram tidas como essenciais para os resultados de melhoria alcançados.

O ciclo de melhoria foi considerado adequado para implementar mudanças e incentivar boas práticas, devendo ser continuado para que se alcance um nível aceitável de qualidade para todos os critérios, inclusive aqueles que, apesar de terem alcançado melhorias significativas, ainda representam problemas importantes de saúde pública, como a subnotificação. Os problemas identificados, bem como os moduladores contextuais do efeito, devem ser considerados em futuras intervenções.
